# Expression of CFTR, a hallmark gene of ionocytes, is downregulated in salivary glands of Sjögren’s syndrome patients

**DOI:** 10.1186/s13075-022-02959-8

**Published:** 2022-12-07

**Authors:** Qi Zhang, Xiuying Lv, Ying Wang, Bin Wang, Yan He, Chubing Chen, Guixiu Shi, Yan Li

**Affiliations:** Department of Rheumatology and Clinical Immunology, The First Affiliated Hospital of Xiamen University, Medical College, Xiamen University, Xiamen, Fujian China

**Keywords:** Sjögren’s syndrome, CFTR, Ionocytes, Pathogenesis

## Abstract

**Introduction:**

The autoimmune exocrinopathy, Sjögren’s syndrome (SjS), is associated with secretory defects in salivary glands. The cystic fibrosis transmembrane conductance regulator (CFTR) of the chloride channel is a master regulator of fluid secretion, but its role in SjS has not been investigated. Our research found a link between CFTR and SjS at the genetic and protein levels, as well as through clinical data.

**Methods:**

We used single-cell RNA sequencing to identify the presence of CFTR in glandular epithelial cells of the human salivary gland (scRNA-seq) and confirmed the difference using immunofluorescence tests in labial glands and clinical data statistics from 44 non-SjS and 36 SjS patients.

**Results:**

The changes of CFTR expression in salivary glands of SjS patients was assessed at both mRNA and protein levels. According to the scRNA-seq analyses, CFTR was the hallmark gene of ionocytes. We firstly identified that SjS had a lower level of CFTR expression in the labial glands than non-SjS at mRNA level.

Using immunofluorescence assays, we also found that CFTR expression was decreased in SjS patients compared to non-SjS. The results of the clinical statistics revealed that CFTR expression was adversely correlated with feelings of dry mouth, lymphocyte infiltration in the labial glands, and certain autoantibodies in serum (antinuclear antibody, anti-Ro/SSA, and anti-La/SSB antibodies).

**Conclusion:**

Those findings above proved an obviously downregulated expression of CFTR in salivary glands of SjS patients and its clinical significance. Dysfunction in CFTR or ionocytes may contribute to SjS pathogenesis and represents a promising therapeutic target.

**Supplementary Information:**

The online version contains supplementary material available at 10.1186/s13075-022-02959-8.

## Introduction

Sjögren’s syndrome (SjS) is an autoimmune inflammatory exocrinopathy that predominantly affects exocrine glands such as the salivary and lacrimal glands [1]. Numerous investigations recently revealed that glandular epithelial cells may be crucial in SjS [2, 3]. Also, it has been shown that structural and functional abnormalities of salivary gland epithelial cells appear to be significant pathogenic elements in the development of SjS and major players in inflammatory cell infiltration [4, 5].

The cystic fibrosis transmembrane conductance regulator (CFTR), which is responsible for the transport of anions such as Cl^−^ and HCO3^−^ across the epithelial cell membrane, is known to be an ATP-binding cassette (ABC) transporter and a cAMP-activated Cl^−^ channel expressed in secretory and absorptive epithelia (involved in the lung, stomach, intestine, kidney, and salivary glands) [6–8]. In addition to its well-described ion transportation function, CFTR plays a crucial role in inflammatory response, according to recent studies [9–11]. CFTR was discovered to be expressed by immune cells other than epithelial cells, such as macrophages and neutrophils [12]. According to research by Heledd et al., CFTR modulators can reduce the excessive proinflammatory response induced by LPS/ATP [13].

The majority of earlier investigations have used mice or rabbits in in-vitro or in-vivo experiments to examine the connection between CFTR and saliva secretion [14–16]. However, there is no concrete proof connecting CFTR to SjS patients. This is the purpose of our study. By using transcriptomic data from single-cell RNA-sequencing (scRNA-seq) and bulk RNA-sequencing (RNA-seq), we initially discovered CFTR as the hallmark gene of ionocytes and also an abnormally expressed gene in SjS. We then used immunofluorescence experiments in labial gland tissues and performed analyses of the correlation between CFTR and various clinical indicators to further investigate the clinical significance of CFTR expression in SjS patients.

## Methods

### Patients and controls

Labial gland samples were collected from SjS patients and controls (non-SjS) with labial gland biopsies in the Department of Rheumatology of the First Affiliated Hospital of Xiamen University between 2017 and 2019. All patients with SjS fulfilled the 2016 ACR/EULAR classification criteria for SjS [17], and malignant tumors, infections, and other rheumatic diseases were excluded. According to the ACR/EULAR criteria, patients without a clinical diagnosis and those whose diagnostic evaluations were insufficient were also excluded. The ethics committee of The First Affiliated Hospital of Xiamen University gave its clearance for this study. Clinical and laboratory data obtained during the collection of labial salivary gland specimens included gender, age, dry mouth symptoms, infiltrated foci, serology, dry eyes symptoms, dry eye test, rheumatoid factor (RF), other autoantibody profiles (ANA, anti-dsDNA, AHA, anti- RNP/Sm, anti-Sm, anti-SSA, anti-Ro-52, anti-SSB, anti-Scl-70, anti-Jo-1, anti-CENP-B, anti-RIB)-which are measured using immunoblotting, erythrocyte sedimentation rate (ESR), α1-acidglycoprotein (AAG), C-reactive protein (CRP), complement 3 (C3), complement 4 (C4), immunoglobulin A (IgA), immunoglobulin G (IgG), and immunoglobulin M (IgM). Detailed clinical characteristics of participants are presented by group in Table 1.

**Table 1:** The clinical characteristics of participants

	Non-SjS	SjS	*p* value
Number (*n*)	44	36	
NO. of women/no. of men	39/5	35/1	0.147
Age, mean (range) years	43.4 (21–64)	44.2 (27–78)	0.969
Dry mouth-positive%	45	74	0.014
Dry eyes-positive%	41	50	0.442
Infiltrated foci-positive%	5	81	0.000
Dry eye test-positive%	33	79	0.065
Serology-positive%	77	86	0.338
RF -positive%	16	35	0.186
ANA-positive%	37	68	0.000
Anti-dsDNA-positive%	13	6	0.300
AHA-positive%	5	9	0.533
AnuA-positive%	5	6	0.874
Anti-nRNP/Sm-positive%	5	3	0.675
Anti-Sm-positive%	3	0	0.351
Anti-SSA-positive%	38	74	0.000
Anti-Ro-52-positive%	38	62	0.006
Anti-SSB-positive%	7	29	0.010
Anti-Scl-70-positive%	3	0	0.351
Anti-Jo-1-positive%	3	0	0.351
Anti-CENP-B-positive%	8	0	0.101
Anti-RIB-positive%	5	0	0.184
ESR mean (range) (mm/h)	26.1 (2–84)	26.5 (3–90)	0.619
CRP mean (range) (mg/L)	2.2 (0.2–20.3)	4.4 (0.16–34.7)	0.812
C3 mean (range) (g/L)	1.0 (0.3–1.4)	1.0 (0.7–1.6)	0.927
C4 mean (range) (g/L)	0.2 (0.1–0.4)	0.2(0.1–0.6)	0.127
IgA mean (range) (g/L)	2.9 (0.6–5.3)	3.1 (1.1–6.7)	0.501
IgG mean (range) (g/L)	15.8 (8.1–32.6)	18,6 (11.0–32.9)	0.023
IgM mean (range) (g/L)	1.4 (0.4–3.4)	1.6 (0.2–7.9)	0.734
CFTR mean (range) mean	32.6 (15.9–61.6)	17.4 (4.7–28.5)	0.000

### Immunofluorescence with labial gland tissue slides

After being fixed in 10% formalin and paraffin-embedded, fresh tissues were cut into sections of 3 μm and sectioned for immunofluorescence. The sections were put in 65 °C drying box for 2 h. Xylene was used to dewax tissue sections, followed by gradient ethanol treatment and citric acid antigen repair. During immunofluorescence, sections were blocked with 5% BSA for 1 h before being incubated with primary antibodies (Anti-CFTR Ponoclonal Antibody, Solarbio, K008791P, diluted 1: 10) at 4 °C overnight, followed by the labeling of secondary antibodies (all 1:400, Alexa Fluor; Invitrogen) for an additional hour at room temperature. The samples were then stained with DAPI and observed under a fluorescent microscope as the final stage (Leica DM2500, Germany). Using imaging software (Image J), an analysis of densitometry and fluorescence intensity was performed. Results were presented as mean optical density values which was equal to an integrated optical density per unit area (mm^2^).

### Hematoxylin and eosin (HE) staining

In accordance with conventional methods, HE staining was applied. Tissue sections were briefly stained with Mayer's hematoxylin (Baso, BA4027, China) for 2 min, followed by rinsing in tap water, after deparaffinization and rehydration. The sections were then dehydrated with graded alcohol followed by cleaning in xylene and staining with eosin solution (Baso, BA4027, China) for 20 s. Finally, microscopic examination and photography of the mounted slides were conducted (Leica DM2500, Germany).

### scRNA-seq analysis

To explore the structures of glandular cells and transcriptomic signatures, we used a scRNA-seq data of human salivary glands (including 5 non-SjS patients) completed by Huang et al. [18]. Analyses of PCA (principal component analysis), UMAP (uniform manifold approximation and projection), tSNE (t-distributed stochastic neighbor embedding), and clustering were performed using Seurat R package. The first 20 principal components (PC) with 0.5 as resolution were clustered using UMAP and tSNE. To further characterize these cell groups, we used the Single R package and surface markers that clearly identify different subsets of glandular epithelial cells. Finally, violin plot displaying the expression of CFTR across cell clusters was generated.

### Salivary gland transcriptomic dataset and differential expression analysis

We compared the levels of CFTR expression in salivary glands between SjS and non-SjS patients with RNA-seq data from GSE173808. This dataset includes a differential expression analysis of the labial glands from 20 non-SjS patients and 39 SjS patients. The expression values were extracted from the transcriptomic data. The downregulation of CFTR between two groups was determined by the value of fold change.

### Statistical analysis

The formula mean ± standard deviation (SD) was applied to continuous variables. For distributions that are not normal, non-parametric tests were performed. Spearman coefficients were calculated to determine if two variables were correlated or not. Statistical analysis was carried out with R software (Version 3.6.1, The R Foundation for Statistical Computing) and SPSS22 software. For the *P*-value to be considered significant, it must be lower than 0.05.

## Results

### scRNA-seq analyses proved the expression of CFTR in salivary glandular epithelium and the existence of ionocytes

**Fig. 1:**
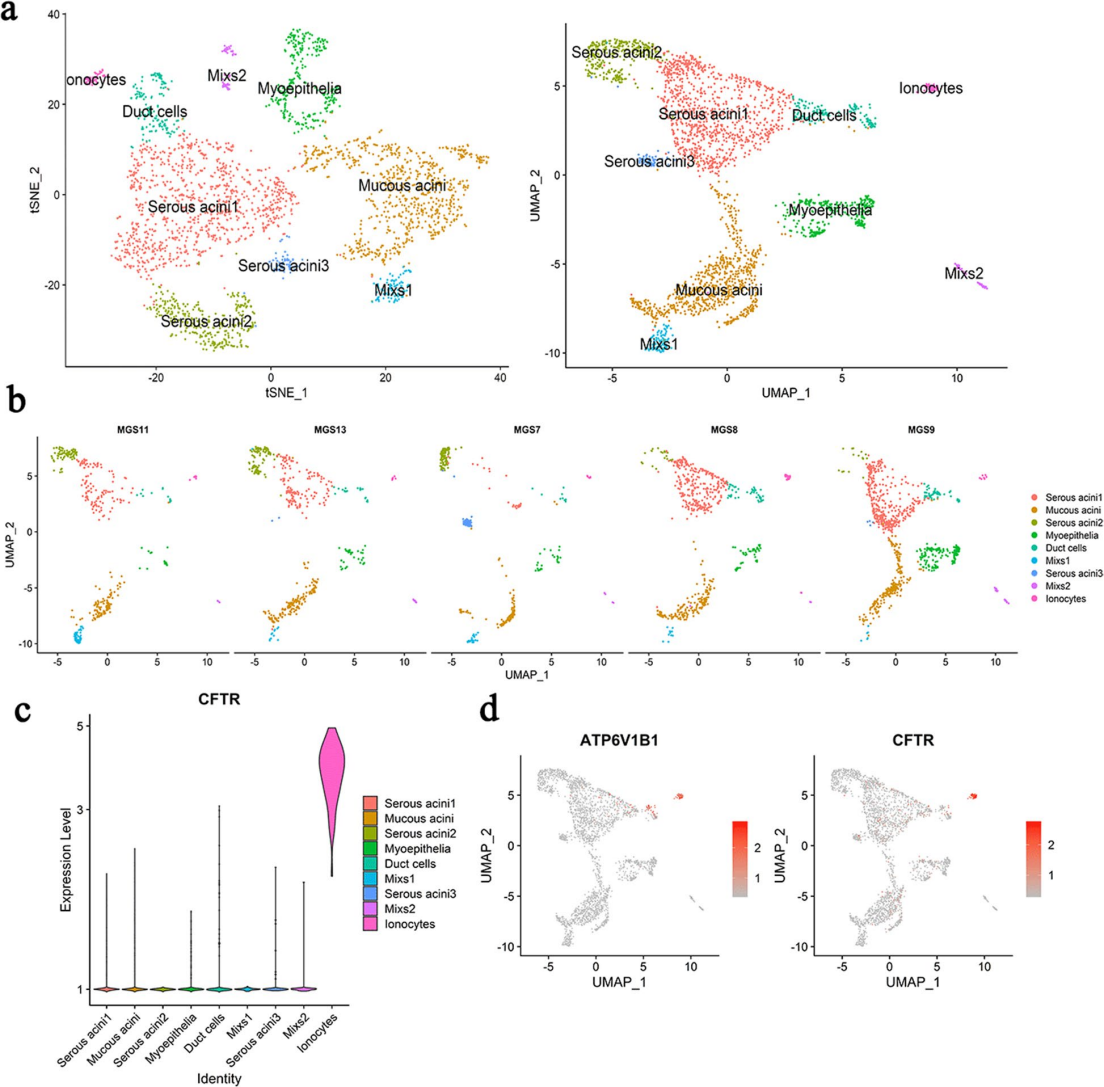
The composition of salivary gland epithelial cell subpopulations was based on scRNA-seq analyses. **a** The subsets of salivary gland epithelial cells clustered by tSNE and UMAP methods. **b** The proportions of the different salivary gland epithelial cell subpopulations in different samples. **c** Violin plot comparing expression levels of CFTR gene in ionocytes and other subsets. The colored area of the violin plot represents the expression values distribution. **d** Feature plot showing the expression pattern of CFTR gene in the salivary gland epithelial cell subpopulations

We explored the composition of salivary gland epithelial cell subpopulations via scRNA-seq analyses (Fig. 1). scRNA-seq analyses divided the glandular epithelial cells into 7 major subgroups in accordance with the surface markers of glandular epithelial cells in the literature [19], including 3 subsets of serous acini cells, a subset of mucous acini cells, a subset of myoepithelial cells, a subset of duct cells, and a subset of ionocytes (Fig. 1).

In order to identify the signature genes of those cell subsets, we used Seurat to determine the genes specifically expressed by each cell subpopulation, that is, genes that are primarily expressed in one specific subpopulation but are largely not expressed in other cell subsets. Ionocytes stood out to us among them, and we discovered its hallmark gene CFTR, which is highly expressed in ionocytes importance. CFTR was highly expressed in ionocytes (Fig. 1c, d).

### Patients with SjS had downregulated CFTR gene expression

Data of CFTR from the matrix effect in the cluster GSE173808 was statistically analyzed using the independent t-test to ascertain whether gene expression difference was statistically significant. To satisfy normality assumptions, the data were initially transformed to a log scale (base 10) (Supplementary Table 1). The difference between the CFTR gene values of the controls (3.578 ± 0.170) and the patients with SjS (3.432 ± 0.220) was 0.146 (95% confidence interval (0.031–0.262)). There was statistically difference between the two teams, and gene expression of CFTR was lower in the SjS group than it is in the control group (*P* < 0.05).

### Reduced CFTR expression in SjS patients compared to non-SjS in human labial gland tissues

**Fig. 2:**
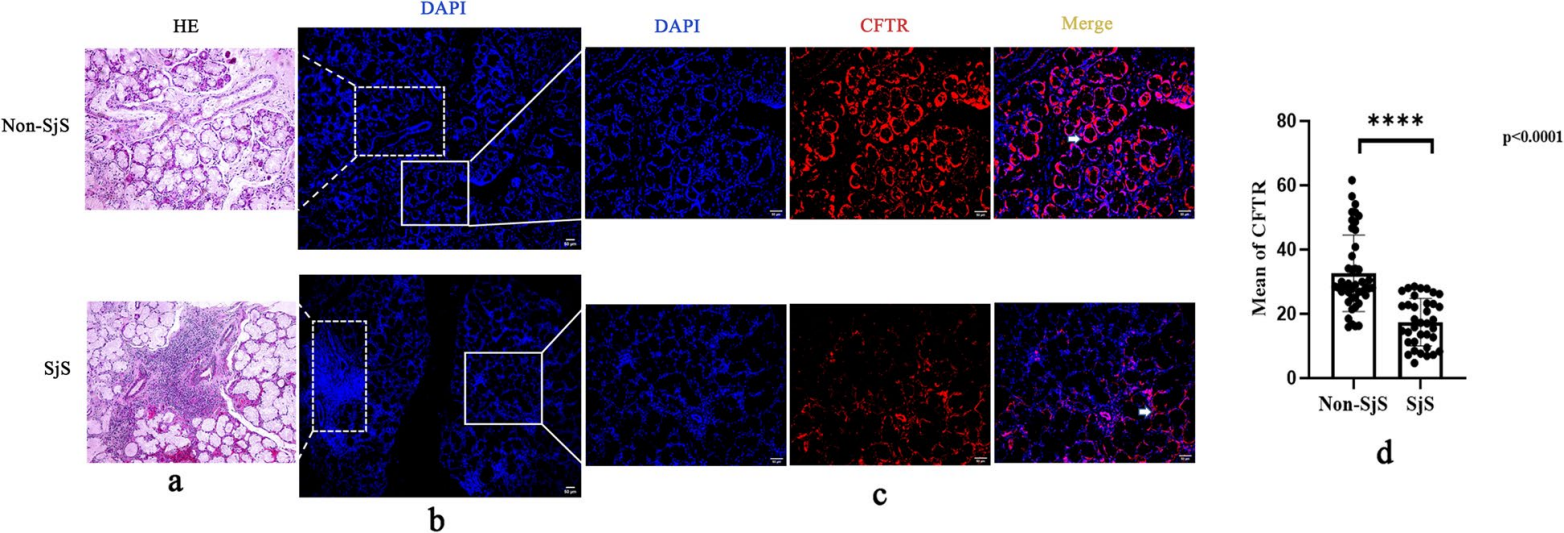
Representative HE-stained and immunofluorescent stained slices for non-SjS and SjS group. Images are × 200 (scale bars, 50 μm) except immunofluorescent stained sections in **b**, which are × 100 (scale bars, 50 μm). Red staining shows CFTR localization and blue DAPI staining shows cell nuclei. **a** Matched slides of labial gland section were stained with hematoxylin and eosin (HE) and the dashed box in **b** indicates the section as in **a**. **b** DAPI staining of labial gland section for non-SjS and SjS group; magnification, × 100. **c** The white solid box in **b** represents the magnified region in the images. White arrowheads point to the expression of CFTR. **d** Comparison of the mean of CFTR expression between non-SjS (*n* = 44) and SjS (*n* = 36) groups. Error bars represent SD. *p* values are based on Mann–Whitney *U* test

Then, using an immunofluorescence technique, we evaluated the expression of CFTR in labial gland tissues from 36 patients with SjS and 44 controls (Non-SjS). Higher magnifications of the white solid boxes in Fig. 2 demonstrated that the expression of CFTR in ductal epithelial cells were significantly decreased in patients with SjS compared with non-SjS (DAPI fluorescence is shown in blue, CFTR immunofluorescence is shown in red). CFTR expression was measured using the Image J image analysis system with mean gray value (IntDen/Area). All data from the sample was analyzed statistically and we discovered that CFTR expression at protein level was markedly reduced in the labial glands of SjS patients (Fig. 2d). The aforementioned findings supported the prior bioinformatic outcomes in which CFTR expression was decreased in SjS patients.

### Downregulation of CFTR was significantly correlated with clinical indicators of SjS patients

**Fig. 3:**
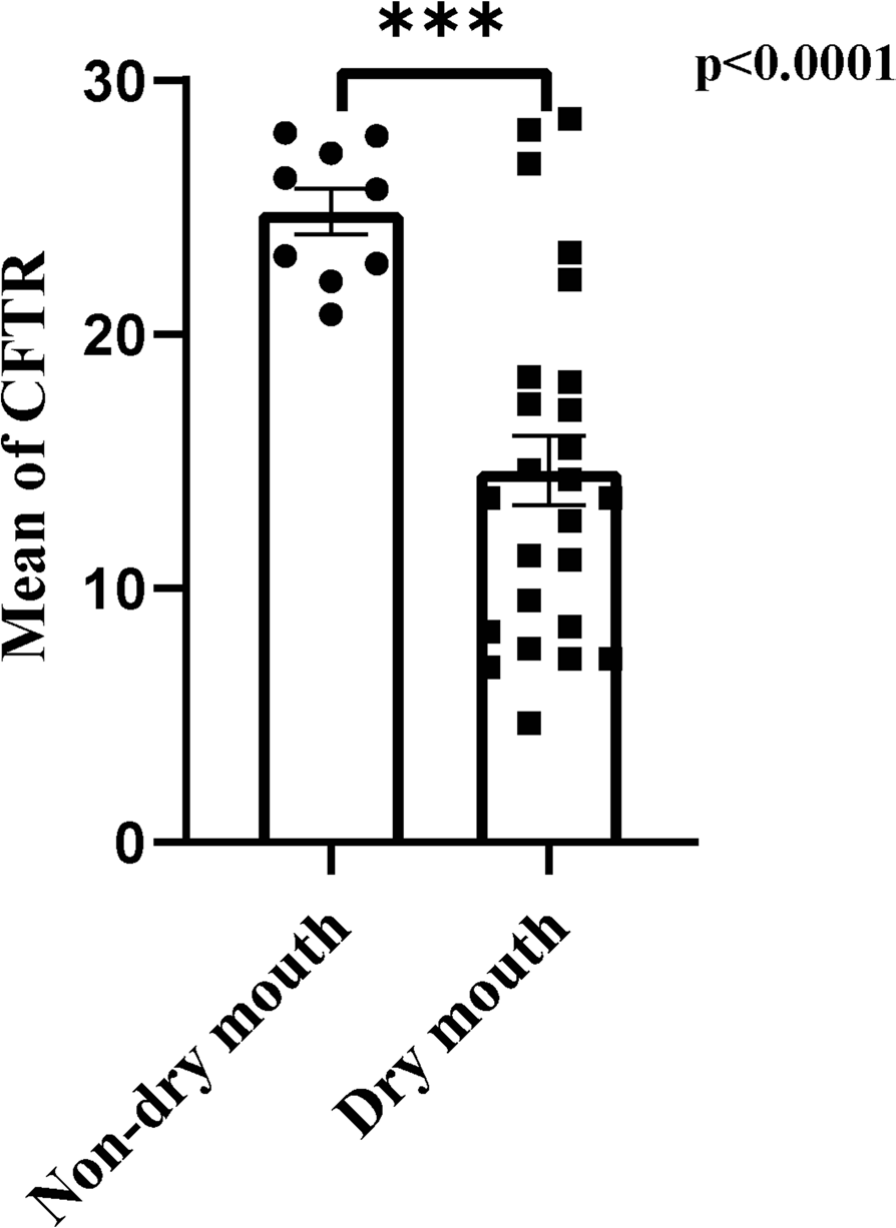
The correlation of CFTR expression in SjS patients with and without dry mouth. Comparing the average CFTR expression across SjS patients with non-dry mouth (*n* = 9) and dry mouth (*n* = 25) groups reveals that CFTR is lower in SjS patients with a system of dry mouth. Error bars represent SEM. *p* values are based on Mann–Whitney *U* test

After gathering clinical data, we performed Spearman correlation analyses to look at the association between the CFTR and clinical parameters (Table 2). It is interesting to note that patients with xerostomia reported lower CFTR gene expression than those without xerostomia. Moreover, there was a tendency for negative associations between the CFTR level and several serum immunological markers as well as the quantity of infiltrated foci in the labial salivary gland (ANA, anti-SSA and anti-SSB). However, there was no discernible relationship between the expression of CFTR and markers of disease activity (except C4), gender, age, dry eye state, dry eye test, and other autoantibody levels (Tables 2, Supplementary Tables 2 and 3). Figure 3 showed SjS patients with dry mouth symptoms had significantly lower CFTR value than those without dry mouth symptoms.

**Table 2:** The associations between the participant's clinical traits, the number of infiltrating foci in their labial salivary gland, and the CFTR expression level

Variable	*r*	*p*
Gender (man/woman)	0.231*	0.039
Age (years)	0.026	0.819
Dry mouth (yes/no)	− 0.485**	0.0001
Infiltrated foci (*n*)	− 0.556**	0.0001
Serology (positive/negative)	− 0.091	0.439
Dry eyes (yes/no)	− 0.174	0.159
Dry eye test (positive/negative)	− 0.217	0.197

^*^Significant at the 0.05 level (2-tailed)

^**^Correlation is significant at the 0.01 level (two-tailed)

## Discussion

SjS is still a matter of speculation regarding the etiology and pathogenesis. It has been suggested that there is a close relationship between these salivary gland epithelial cells and the development of SjS [20]. The CFTR-expressed ionocyte is a recently discovered epithelial cells and has key function in mucus secretion [21, 22]. There was no report in the literature assessing its function in SjS. In this study, we utilized scRNA-seq analyses and immunofluorescence assays to uncover the roles of CFTR and/or ionocytes in SjS. Our findings proved an obviously downregulated expression of CFTR in salivary glands of SjS patients and its clinical significance.

More than 90% of SjS patients complain of symptoms including xerostomia that are linked to salivary gland dysfunction [23]. As the foundation for saliva secretion, the duct of the salivary gland absorbs both Na^+^ and Cl^−^ and secretes K^+^ and HCO3^−^. Apart from neuronal regulation [24], a number of other elements, such as CFTR regulation, have recently come into focus as they relate to salivary secretion. By controlling the activity of many transporters in the luminal membrane of the duct, CFTR serves as a major regulator of ductal fluid and electrolyte secretion in addition to Cl^−^ and HCO_3_^−^ channel activity. CFTR also interacts with AKAPs, kinases, SNARE proteins, and phosphatases, which is important for ductal secretion [7, 25].

As is well known, the majority of cystic fibrosis (CF) cases result from a CFTR mutation. Recently, the existence of a small number of ionocytes in the respiratory epithelium that express CFTR at a high level has been documented in the literature [26]. The fact that CFTR is also present in other epithelial cells but is particularly prominent in ionocytes is still remarkable [22, 26, 27]. According to Zeng M. et al., CFTR potentiators (VX-770) can restore the expression level of CFTR in the salivary gland duct epithelium, which will reduce the inflammatory reaction and tissue damage [28]. Additionally, transgenic overexpression of CFTR in the salivary gland duct epithelium can play a similar effect [28]. Recent research by Flores AM et al. demonstrated that CFTR potentiators improved tear production in a mouse model of dry eyes [29]. Furthermore, there have been some clinical situations where individuals with faulty CFTR mutations have developed SjS [30–32]. These results indicated a critical role for highly expressed CFTR in sustaining fluid discharges in both salivary and lacrimal glands and that dysfunction of CFTR may play a role in the pathophysiology of SjS. Transcriptomic data and the immunofluorescence microscopy outcomes in our study revealed that a subpopulation of ionocytes (including CFTR) that was isolated from epithelial cells of the human salivary glands was downregulated in patients with SjS. It was indicated that people with SjS might benefit therapeutically by targeting CFTR defect.

Aside from dry mouth and dry eyes, other clinical signs of SjS include arthralgia, myalgia, purpura, peripheral neuropathy, abnormal blood indicators, and more [33]. Typically, each patient has a unique set of clinical symptoms. Treatment frequently benefits from further patient classification based on different clinical presentations. Given that there is a greater correlation between the expression of CFTR and dry mouth system in SjS patients than non-SjS, the findings we above may reveal that CFTR is connected to the development of the SjS condition. Medication that targets the CFTR may be very beneficial for patients who only or predominantly have symptoms of dry mouth.

However, many aspects of CFTR remain a mystery, including their regulation, signaling, environmental sensing, and potential interaction with the nervous system. CFTR stimulation may has the advantage of treating an early event in the pathophysiology of SjS when compared to immunosuppressive methods. Thus, the development potential of CFTR activators as the first-ever dry mouth treatment is supported by our data.

According to the above study, CFTR is dramatically decreased in the salivary tissues of SjS patients. Additionally, it has been proposed that CFTR dysfunction or a deficiency may have contributed to the etiology of SjS. To identify the precise mechanism of action, more study is necessary.

## Supplementary Information

**Additional file 1:** **Supplementary Table 1.** Results for the normality tests. **Supplementary Table 2.** The relationship between the CFTR gene and SjS-related immunological indices. **Supplementary Table 3.** The connection between CFTR gene and some definitive serum biomarkers associated with disease activity in SjS.

## Data Availability

All data generated or analyzed during this study are included in this published article.
