# Supplementary material for: Expression of CFTR, a hallmark gene of ionocytes, is downregulated in salivary glands of Sjögren’s syndrome patients
Source: Arthritis Res Ther. 2022 Dec 7;24:263. doi: 10.1186/s13075-022-02959-8 (PMC9727938; doi:10.1186/s13075-022-02959-8)
Supplement: Supplementary file 1 — Additional file 1: Supplementary Table 1. Results for the normality tests. Supplementary Table 2. The relationship between the CFTR gene and SjS-related immunological indices. Supplementary Table 3. The connection between CFTR gene and some definitive serum biomarkers associated with disease activity in SjS. [file 13075_2022_2959_MOESM1_ESM.docx]

**Supplementary Table 1 Results for the normality tests**

| Dependent variable |  | Kolmogorov-Smirnova | | |  | Shapiro-Wilk | | |
| --- | --- | --- | --- | --- | --- | --- | --- | --- |
|  |  | Statistic | df | Sig. |  | Statistic | df | Sig. |
| Expression of CFTR | con | 0.088 | 19 | 0.200^*^ |  | 0.980 | 19 | 0.941 |
|  | SjS | 0.077 | 38 | 0.200^*^ |  | 0.988 | 38 | 0.943 |

**Supplementary Table 2 The relationship between the CFTR gene and SjS-related immunological indices**

| Variable | r | p |
| --- | --- | --- |
| RF (positive/negative) | -0.131 | 0.330 |
| ANA(n) | -0.358^**^ | 0.001 |
| Anti-dsDNA(n) | 0.116 | 0.335 |
| AHA (n) | 0.049 | 0.688 |
| AnuA (n) | -0.001 | 0.991 |
| Anti-nRNP/Sm (n) | -0.026 | 0.831 |
| Anti-Sm (n) | 0.105 | 0.384 |
| Anti-SSA (n) | -0.306** | 0.007 |
| Anti-Ro-52 (n) | -0.146 | 0.209 |
| Anti-SSB (n) | -0.259* | 0.024 |
| Anti-Scl-70 (n) | 0.076 | 0.530 |
| Anti-Jo-1 (n) | 0.134 | 0.265 |
| Anti-CENP-B (n) | 0.161 | 0.181 |
| Anti-RIB (n) | 0.004 | 0.973 |

* Significant at the 0.05 level (2-tailed).

** Correlation is significant at the 0.01 level (two-tailed).

**Supplementary Table** **3** **The connection between CFTR gene and some definitive serum biomarkers associated with disease activity in SjS**

| Variable | r | p |
| --- | --- | --- |
| ESR (mm/h) | 0.214 | 0.210 |
| AAG (g/L) | 0.003 | 0.985 |
| CRP (mg/L) | 0.129 | 0.271 |
| C3 (g/L) | 0.219 | 0.059 |
| C4 (g/L) | 0.242* | 0.037 |
| IgA (g/L) | -0.154 | 0.197 |
| IgG (g/L) | -0.165 | 0.157 |
| IgM (g/L) | 0.032 | 0.788 |

* Significant at the 0.05 level (2-tailed).

** Correlation is significant at the 0.01 level (two-tailed).
